# Experimental Research into the Uniaxial Compressive Strength of Low-Density Reef Limestone Based on Image Recognition

**DOI:** 10.3390/ma16155465

**Published:** 2023-08-04

**Authors:** Xiaoqing Wei, Yi Luo, Yuhang Tao, Xinping Li, Fei Meng

**Affiliations:** 1 Sanya Science and Education Innovation Park, Wuhan University of Technology, Sanya 572024, China; xqwei@whut.edu.cn (X.W.); feimeng@whut.edu.cn (F.M.); 2 School of Civil Engineering and Architecture, Wuhan University of Technology, Wuhan 430070, China

**Keywords:** low-density, reef limestone, image recognition, uniaxial compressive strength

## Abstract

Low-density reef limestone is widely distributed in tropical oceans; exploring its mechanical properties is of significance to practices in marine foundation engineering. In this research, laboratory experiments on low-density reef limestones with two different types of porous structures were conducted using image recognition methods to study the special mechanical properties of low-reef limestone. $\bar{S}$ was defined as the parameter quantifying the pore geometry, and the calculation method of $\bar{S}$ was optimized based on image recognition data. Finally, the influencing factors of uniaxial compressive strength (UCS) of low-density reef limestone were analyzed, and a modified formula considering pore structure was proposed. The results indicate the following: Image recognition methods were used to determine feasibility and convenience of capturing 2D pore geometric information of specimens. The optimization method of $\bar{S}$ is conducive to improving automatic image recognition accuracy. Low-density reef limestones with different porous structures show small difference in porosity and density, while they exhibit large differences in pore sizes and UCS. The UCS of low-density reef limestone is found to be jointly influenced by pore structure and density (it increases with the decrease in parameter $\bar{S}$ and increase in density). The results may provide help for those investigating the mechanical properties of reef limestone and practices in marine foundation engineering.

## 1. Introduction

With the advances in technology and human exploitation and utilization of maritime resources, the number of construction projects on coral reefs has increased, and widespread concerns about the geological properties of coral reef have arisen [1,2]. Coral reefs are mostly developed in tropical marine areas and are characterized by a binary structure along the vertical direction. A reef’s shallow layer mainly comprises coral sand, and its deep part is composed of reef limestones, accompanied by karst cave and erosion surfaces [3,4,5,6].

Reef limestone is a type of rock with complicated sources. The remains of reef-building organisms including coral, foraminifera, and coral algae and the remains of lagoon sediments are major components of the skeleton of reef limestone. Reef limestone is mainly made up of minerals including aragonite, calcite, and dolomite, and its diagenesis is primarily subjected to effects of compaction, cementation, and corrosion [5,7,8].

Due to the complex constituents and diagenetic process, the mechanical properties of different types of reef limestone vary significantly. The uniaxial compressive strength (UCS) of reef limestone is a basic parameter for evaluating mechanical properties, which has been widely studied. Zhu et al. [9,10,11] examined bioclastic limestone and found that the density and degree of cementation are key parameters controlling the UCS of bioclastic limestone, while porosity exerts little influence on the UCS. Wang [8] and Wang et al. [12] performed laboratory mechanical tests to measure the physico-mechanical properties of reef limestone in the Nansha Islands. Their results indicated that reef limestone under uniaxial compression underwent tensile failure along the growth line, with significant residual strength remaining after tensile failure. Pappalardo [13] conducted laboratory tests on reef limestone sampled in the south of the Peloritani Mountains. The results of each test exhibited significant discreteness, among which the UCS has a maximum value of 112 MPa and minimum value of 15.18 MPa, indicating the UCS is linearly correlated with elastic modulus and the velocity of a longitudinal wave. Amr [14] found that there is good linear correlation between the UCS and elastic modulus of calcareous sandstone in Dubai. Ma et al. [15] compared uniaxial compressive properties of reef limestone at different depths and found that deep reef limestone had an average UCS 4.78 times that of shallow reef limestone, while its physical properties are superior to those of reef limestone. Liu et al. [16] found that the density of reef limestone is linearly correlated with porosity by experimentally investigating reef limestone with different structures, while two types of reef limestone saturation with lower density were negatively correlated with UCS and porosity. Moreover, Liu et al. [17] applied the lateral strain response method (LSR) method to the triaxial compression test and found that under a confining pressure of 0–8 MPa, the crack initiation stress of the specimen is estimated to be 52–69% of the peak compressive strength. Wu et al. [18,19,20] performed a series of laboratory tests to perform in situ scanning of specimens under different stress states with regard to understanding the damage evolution characteristics, microscopic mechanism of crack propagation, and quality evaluation of CRL under uniaxial compression.

According to the aforementioned analysis, the UCS characteristics of reef limestone have been extensively investigated: it is noteworthy that some of these studies have explored reef limestone samples with large density, e.g., reef limestone samples had a minimum density of 1.33 g/cm^3^ and a maximum density of 2.7 g/cm^3^. The mechanical properties of reef limestone samples with a lower density are rarely reported.

For the literature on the UCS characteristics of low-density reef limestone, Burton et al. demonstrated an intriguing phenomenon: for *Diploria*, as the one of three main types of coral in sites around Barbados, the young reef limestones developed had a minimum density of only 0.53 g/cm^3^, but achieved a relatively high UCS value in a mechanical test, which are largely different from results pertaining to other reef limestones with high densities in the same test. Burton et al. inferred that this type of reef limestone exhibits an especially efficient calcite structure. There is no other in-depth research relating to such topics being reported.

Low-density reef limestone underwent slight compaction and cementation owing to their late diagenesis, showing significant primary porous structures. Hence, the study of physical properties for low-density reef limestone as a porous material warrants consideration in terms of the influence of porous structures [21]. Zeng et al. [22] used digital image analysis theory to digitize the shape characteristics of coral particles, and established a simulation model of the dense packing of particles method. Liu et al. [23] analyzed the pore–throat networks inside representative elementary volumes of marine carbonates samples using X-ray micro-computed tomography (CT) scanning technology, and found that the type of rock and the porosity seem to have little effect on the shapes of the pores and throats. Zhang et al. [24] found that the strength of coral aggregate is dependent on the maximum pore size and porosity of small pores (0~0.2 mm) by combining CT scan and uniaxial compression test results. Wei et al. [25] reviewed the external and internal pore structure of coral aggregate and found that the exponential function can be used to describe the changing trend of the compressive strength of coral aggregates with porosity. Wang et al. [26] studied the pore structure characteristics and seepage characteristics of two kinds of coral reef limestone by combining computed tomography (CT) scanning technology and graph theory. 

Although use of X-ray CT can acquire detailed characteristic data of porous structures for reef limestone [15,27,28], the cost associated with such testing is high. Image recognition technology demonstrates advantages including high efficiency and cost-effectiveness; it has thus been broadly applied in many fields, as shown in Table 1. Thus, in this study, an image recognition technique was chosen to research the characteristics of pore geometry for low-density reef limestone.

As the current factors influencing the UCS of low-density reef limestone remain unclear, this research quantified porous structures of low-density reef limestone by means of an image recognition technique based on testing results. On this basis, by referring to the research methods of other rocks [35,36,37], the influences of basic physical properties and porous structures on the UCS of low-density reef limestone were explored.

## 2. Experimental Work

### 2.1. Test Specimens

**Figure 1:**
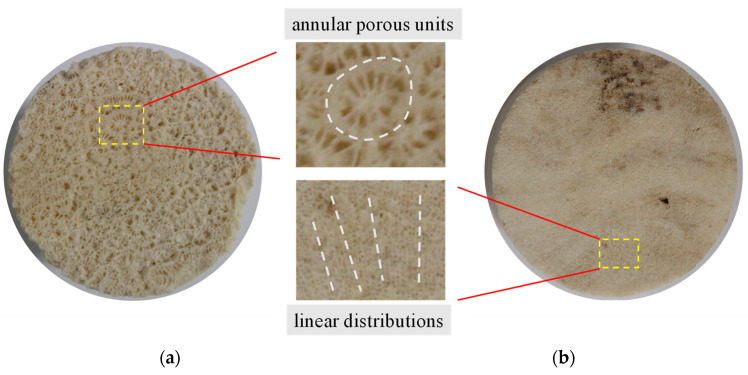
Low-density reef limestone specimens. (**a**) S-type; (**b**) M-type.

The specimens used in the experiment were taken from the shoal of a tropical sea. Referring to the specifications suggested by the International Society for Rock Mechanics and Rock Engineering (ISRM) [38], the samples were subjected to processes such as coring, cutting, and grinding and finally made into *Φ* 50 × 100 mm standard cylindrical specimens. In this test, two types of specimens with largely different porous structures were used as the control group, which were labelled S and M. The morphologies of two types of specimens are illustrated in Figure 1.

As shown in Figure 1, the morphologies of the porous structures for the two types of reef limestone are visible to the naked eye. Among these specimens, several annular porous units can be seen on the surface of S-type specimens; the pores within the units present a radiating distribution. The morphology of each pore is shown to be spindle-shaped. For M-type specimens, there are biologically eroded holes and wave-shaped growth lines found on their surface. Through partial enlargement, dot-shaped pores are characterized by linear distributions. Overall, the single pore size of S-type specimens is much greater than that of M-type specimens. In addition, the structural characteristics of each pore within the same type of specimens are akin to that of their geometric sizes.

### 2.2. Pore Image Recognition of Specimens

#### 2.2.1. Software

In this research, Image J 1.52i software, as a JAVA-based open-source image analysis software developed by the National Institute of Mental Health, in the US was adopted to identify the images of the pores for the specimens. In addition to fulfilling basic processing of images, Image J can be used to perform statistical analysis of specific image pixel zones, including number, size, and position. It has been widely applied in immunohistochemical quantification, grey-scale analysis of Western blot assay results, and cell counting [39].

#### 2.2.2. The Process of Image Recognition

**Figure 2:**
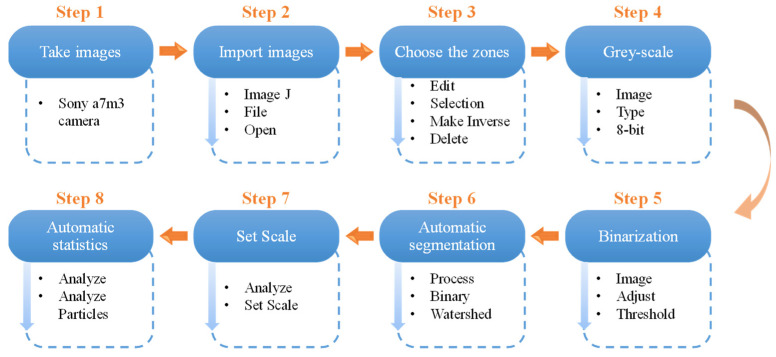
Image recognition process of pores.

To acquire the data of pore geometry for reef limestones considering comparison of image recognition results for different reef limestone specimens, image recognition of all specimens was realized according to the process in Figure 2.

As shown in Figure 2, solid boxes refer to the name of each step, and the contents in dashed boxes describe processing steps. In Step 1, to ensure image definitions, a Sony a7m3 camera with a Tamron A071 28–200 mm lens is adopted. In Step 2, a camera bracket and built-in flash are used in photographing process to reduce the difference in photographic illumination angles for different specimens. In addition, Image J software was used to import images as photographed. In Step 3, the box-selection tool is employed to choose the zones to be processed and then using Make Inverse—Delete, other zones can be deleted; thereafter, the images are subjected to grey-scale processing in Step 4 and binarization in Step 5, and the separation between porous zones and skeleton zones is realized. When pore density is large, a phenomenon whereby pores overlap and pores lie close to each other arises: the Watershed Algorithm is used to automatically recognize the overlapping parts and separate two different porous zones in Step 6. In Step 7, before automatic statistical analysis of the pores, it is necessary to measure the ratio of the length of a line to the length of the actual line using a line tool. By doing so, a measuring scale is established. In Step 8, Analyze Particles can be used to realize the automatic statistics of porous zones in images. Furthermore, data including area, center, and perimeter of each pore are exported.

As the grey-scale interval of image binarization in Step 5 is valued using manual selection, it is largely influenced by subjective consciousness; therefore, a reasonable grey-scale interval is selected by comparing pore image recognition effects at different grey-scale intervals to reduce the influence of subjectivity on interval selection.

#### 2.2.3. Quantification of Pore Geometry

Although reef limestones, as typical porous media, demonstrate multiple types of pore structures, the pore structures in a given zone are similar. This is because the reef limestones in the zone originated from the same type of reef-building organisms. As image recognition results obtained are 2D geometric data pertaining to the pore structures in a given specimen, for convenience of analysis, this research used the area as the parameter quantifying geometry of individual pores by neglecting shape differences in different single pores. The average area was used as the parameter quantifying the pore geometric characteristics at specimen level. Thus, $\overset{-}{S}$ is defined as the parameter characterizing pore geometry and can be calculated as follows:

(1) $$\overset{-}{S}=\frac{\sum_{i=1}^{n}S_{i}}{n}$$

where n denotes the number of pores in image recognition results, $S_{i}$ refers to the area of the ith pore, and $\overset{-}{S}$ represents the average pore area of image recognition zones.

### 2.3. Experimental Design

The experiment was conducted following conventional UCS research and experimental process of rock. The experimental steps are as follows: (1) Porosity and density of reef limestone specimens were measured in the laboratory. (2) The pores of each specimen are identified through image recognition according to the method described in Section 2.2.2. Based on this, the optimal grey-scale interval of image binarization (seen in Step 5 of the process of image recognition in Section 2.2.2) can be ascertained. (3) The optimal grey-scale interval obtained in the last step is used to perform pore image recognition on all specimens; afterwards, the values of pore geometric parameter $\overset{-}{S}$ for different specimens are calculated. (4) Uniaxial compression tests were conducted to determine UCS values of specimens in the Hainan Deep-Sea Technology Innovation Center, China. The apparatus used was an JD-1002A Computer Control Electro-Hydraulic Servo-motor Universal Testing Machine (Jiedong Testing Equipment Co., Ltd., Dongguan, China) at a loading rate of 0.5 mm/min.

## 3. Experimental Results and Analysis

### 3.1. Influences of the Process Parameters on Image Recognition Performance

In this research, image binarization aims to distinguish between porous zones and skeleton zones in images to lay a foundation for automatic statistics of pores. In the following part, the manual recognition results are taken as the benchmark, and the image recognition performances at different grey-scale intervals were compared to determine the optimal grey-scale interval in the experiment.

**Figure 3:**
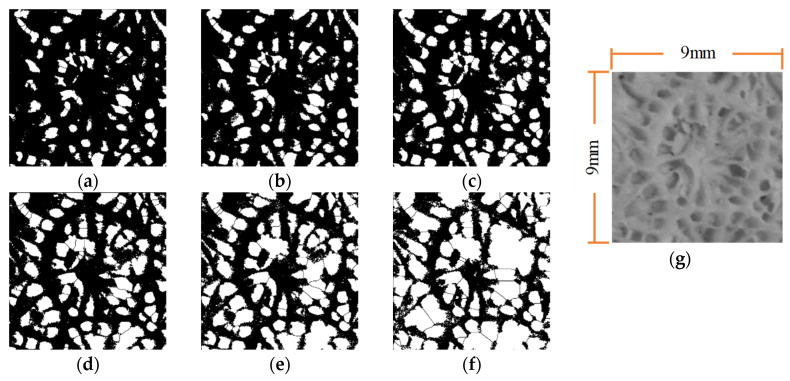
The comparison of pore image recognition results at different values of *A.* (**a**) *A* = 115; (**b**) *A* = 120; (**c**) *A* = 125; (**c**) *A* = 125; (**e**) *A* = 135; (**f**) *A* = 140; (**g**) Gray-scale image.

A reef limestone specimen (S-1) was chosen to be processed following the steps in Section 2.2.2. For the sake of clarity, a square zone measuring 9 mm × 9 mm was used as the zone to be processed. The grey-value of pixels in grayscale images is distributed within an interval of 0–255, and we let parameter *A* be the upper limit of the selected grey-scale interval to be processed in the image binarization, while parameter *B* is the lower limit thereof. Testing and analysis show that when *B* is below 40, and *A* is within the range from 115 to 140, the automatic image recognition performance of pores is better. For this reason, the image recognition results when *B* is 25 and *A* is set to 115, 120, 125, 130, 135, and 140 were compared with the original grey-scale image (Figure 3).

As illustrated in Figure 3a–f, white zones represent pores; while black zones denote skeleton zones. Figure 3 indicates that, as *A* increases, image recognition performance is shown to be good at first and then becomes poor; when *A* is small (Figure 3a), the recognition zone for a single pore is smaller than the actual recognition zone, while smaller pores are not identified; when *A* is large (Figure 3f), the recognition zone for a single pore is larger than the actual recognition zone and the skeleton zones in original images are occupied, a phenomenon whereby a large area of pore boundary overlapping occurs, largely affecting image recognition accuracy and leading to a poor automatic boundary segmentation effect when using the Watershed algorithm.

**Figure 4:**
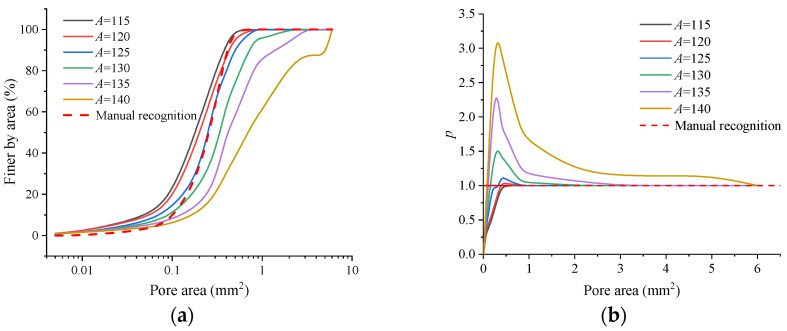
Comparison between image recognition results at different values of *A* and manual recognition results. (**a**) The cumulative curves of the pore area distribution; (**b**) Comparison of image recognition performances.

Image recognition data obtained at different values of *A* and manual recognition data were used to plot cumulative curves of the pore area distribution (Figure 4a). The parameter *p* is defined as the ratio of the image recognition value to the manual recognition value. The closer *p* is to 1, the better the image recognition performance; *p*-value curves for different values of *A* are plotted (Figure 4b).

As can be seen from Figure 4, with the increase in *A*, the cumulative curves of the pore area distribution gradually deviate to the right; the proportion of small pores in the total number of pores decreases (so the proportion of large pores increases). This finding arises because, as *A* increases, the recognition zone for a single pore is gradually enlarged; when *A* = 125, image recognition results can match those achieved using manual recognition; hence, in the following research, 25~125 is chosen as the grey-scale interval selected for image binarization.

### 3.2. Optimization of the Method of Calculation of $\overset{-}{S}$

**Figure 5:**
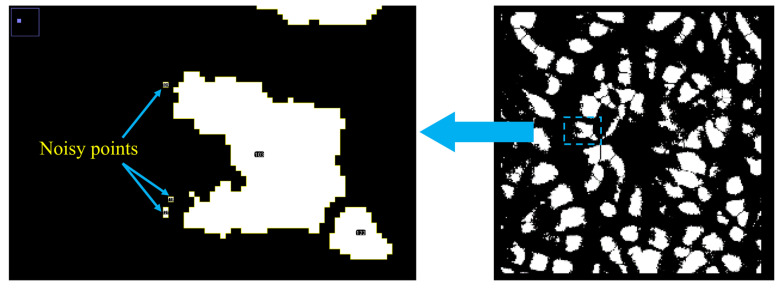
The noisy points in image recognition results.

Figure 4a shows that, compared with manual recognition results, image recognition results in the interval from 0.1 mm^2^ to 1 mm^2^ match data achieved via manual recognition, while in the interval from 0 mm^2^ to 0.1 mm^2^, the degree of fit between the image recognition results and manual recognition results is poor. Using the particle tracking function in Image J, it is found that in automatic statistical analysis, the statistics pertaining to noisy points with small size in images can be extracted (Figure 5). This leads to poor fitting between image recognition results in the case of small pores and manual recognition results.

Further analysis implies that the area of a single noisy point is much smaller than the area of a single pore (there are order-of-magnitude differences between the two). To reduce the error caused by noisy points and consider that areas of different pores in a given specimen are of the same order of magnitude, image recognition results were processed following the steps below to optimize the interval used to calculate the parameter $\overset{-}{S}$.

(1) According to the difference in the order of magnitude, first-level statistical intervals are classified;
(2) In each first-level interval, 10 second-level statistical intervals are proportionally classified;
(3) The first-level statistical interval in which all porous areas account for the largest proportion of the total area of the image and all of its second-level statistical intervals are non-zero is seen as an effective interval;
(4) According to Equation (1), the data related to the effective interval are calculated to deduce the pore geometric parameter $\overset{-}{S}$.

The effect of this optimization method is verified below. Taking images chosen in Section 3.1 as examples, and comparing them with manual recognition results, the raw data of image recognition results and the $\overset{-}{S}$ values calculated based on optimized data are listed in Table 2.

As can be seen from Table 2, the original data of pore automatic image recognition are optimized. In comparison with manual recognition results, total pore area deviation is reduced to 0.891 mm^2^, optimized from 3.701 mm^2^ beforehand; the deviation in number of pores is decreased to 22, optimized from 148 beforehand; the deviation of the parameter $\overset{-}{S}$ is reduced to 0.0707, optimized from 0.1 beforehand. It should be noted that the parameter $\overset{-}{S}$ (as optimized) has the same order of magnitude as that obtained through manual recognition. In summary, the total pore area, number of pores, and the parameter $\overset{-}{S}$ which are optimized better conform to the manual recognition results. This optimization method is conducive to improving automatic image recognition accuracy. Hence, in the following research, the parameter $\overset{-}{S}$ is calculated based on optimized data of automatic image recognition results.

### 3.3. The Influence of Pore Structure on Testing Results

The results for each specimen are summarized in Table 3.

**Figure 6:**
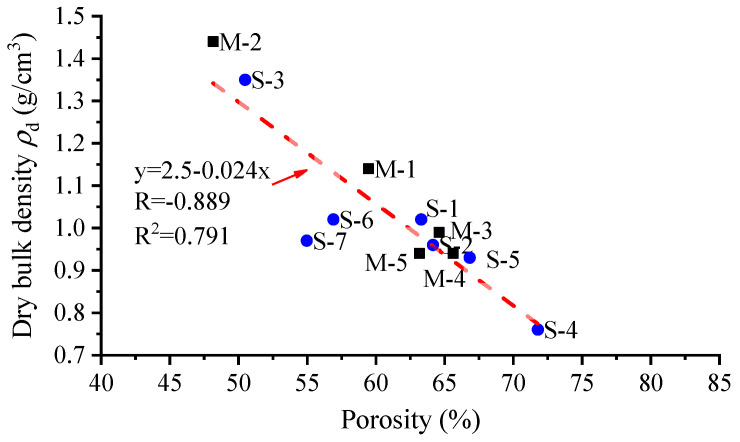
Correlation between porosity and density of specimens.

As can be seen from Table 3, the maximum porosity of S-type reef limestone is 71.79% and the minimum porosity is 50.48%, while the maximum density is 1.35 g/cm^3^ and the minimum density is 0.76 g/cm^3^. The maximum pore geometric parameter $\overset{-}{S}$ is 0.424 mm^2^, while the minimum value of $\overset{-}{S}$ is 0.266 mm^2^, which are both within the same order of magnitude. Moreover, the maximum UCS is 4.730 MPa, and the minimum value is 1.467 MPa. The maximum porosity of M-type reef limestone is 65.61% and the minimum porosity is 48.16%, while the maximum density is 1.44 g/cm^3^ and the minimum density is 0.94 g/cm^3^. The maximum value of $\overset{-}{S}$ is 0.040 mm^2^, while the minimum $\overset{-}{S}$ is 0.030 mm^2^, which are also both within the same order of magnitude. In addition, the maximum UCS is 15.160 MPa, and the minimum UCS is 7.686 MPa. Comparing the testing results of S and M-type specimens, it was found that low-density reef limestones with different porous structures show smaller differences in terms of porosity and density, while they differ significantly in terms of parameter $\overset{-}{S}$ and UCS. Based on the porosity and density correlation graphs drawn using the data in Table 3, data points could be linearly fitted with results (Figure 6).

As shown in Figure 6, generally, there is a linear correlation between the porosity and density of low-density reef limestone specimens. The upper and lower limits of the porosity distribution interval for S-type low-density reef limestone are greater than those of M-type specimens; the upper and lower limits of the density distribution interval for S-type specimen are both smaller than those of M-type specimens. Among all data points, data points for specimens S-6 and S-7 deviate significantly from the regression line because the size of a single pore in the S-type specimens is greater, so water leaks from the pore when measuring the saturated weight of the specimens, leading to a lower saturated weight, and thus a lower porosity.

### 3.4. Analysis of Factors Influencing UCS

**Figure 7:**
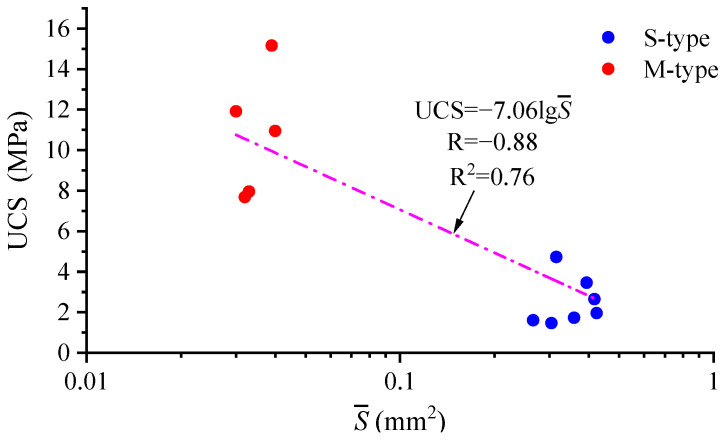
$\overset{-}{S}$-UCS correlation for low-density reef limestone.

According to Section 3.3, it is found that porosity is linearly related to density, and the pore geometric parameter $\overset{-}{S}$ and density $\rho_{d}$ are only considered in the analysis of factors influencing the UCS. The $\overset{-}{S}$-UCS correlation graph for low-density reef limestone is plotted, as shown in Figure 7.

Given that there are order-of-magnitude differences in parameter $\overset{-}{S}$ for two different types of reef limestone, the $\overset{-}{S}$ axis in Figure 7 is logarithmic. As illustrated in Figure 7, there are significant differences in the UCS and $\overset{-}{S}$ between S-type and M-type low-density reef limestones: the M-type reef limestone with a smaller $\overset{-}{S}$ has a higher UCS. The linear fitting results of the UCS and lg$\overset{-}{S}$ are displayed as follows:

(2) $$\mathrm{UCS}=-7.061\;lg\overset{-}{S},\;R=-0.88,\;R^{2}=0.76$$

**Figure 8:**
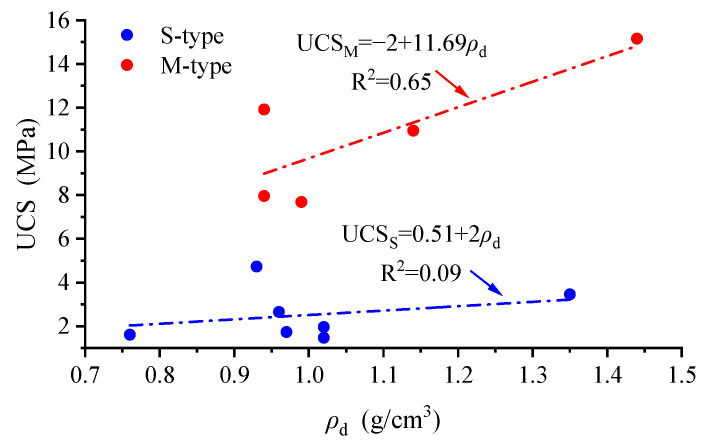
The $\rho_{d}$ -UCS correlation for low-density reef limestone.

Based on the results for the goodness of fit, there is an unfavorable linear correlation between the UCS and lg$\overset{-}{S}$. The graph for the $\rho_{d}$-UCS correlation was plotted (Figure 8).

Figure 8 indicates that UCS generally increases with increasing $\rho_{d}$, and M-type reef limestone has the greater UCS (and it increases more rapidly). The data for two types of low-density reef limestone in Figure 8 were linearly fitted, with results as shown below:

(3) $${\mathrm{UCS}}_{S}=0.51+2\rho_{d},\;R^{2}=0.09$$

(4) $${\mathrm{UCS}}_{M}=-2+11.69\rho_{d},\;R^{2}=0.65$$

The subscripts of the UCS in Equations (3) and (4) refer to the types of low-density reef limestones. Judging from the degree of fit obtained, there are poor linear correlations between the density and UCS of the two types of low-density reef limestones, among which S-type specimens show the poorest linear correlation for the density and UCS. Based on Figure 7, it is inferred that the UCS and density for reef limestone specimens with a more concentrated $\overset{-}{S}$ distribution exhibit stronger linear correlation.

Referring to research by Burton et al. [7], when the density of reef limestone is large, the UCS has favorable linear correlation with density, that is, the UCS and density are related; thus,

(5) $$\mathrm{UCS}=C_{1}+C_{2}\rho_{d}$$

where $C_{1}$ and $C_{2}$ are fitting constants.

According to the aforementioned research, when reef limestone has a low density, there is a poor linear correlation between the UCS and density; meanwhile, the UCS shows a better linear correlation with lg$\overset{-}{S}$. To introduce the influence of pore structure, Equation (5) was modified based on the principle of separation of variables.

(6) $$\mathrm{UCS}=\left(C_{1}+C_{2}\rho_{d}\right)lg\overset{-}{S}$$

**Figure 9:**
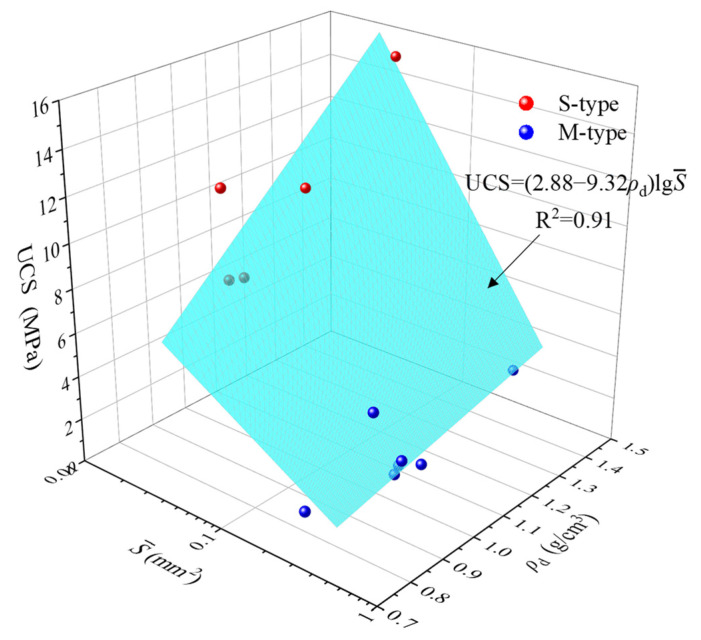
The $\overset{-}{S}$-$\rho_{d}$-UCS correlation for low-density reef limestone.

The test data thus obtained were used to verify the fitting performance of Equation (6). The $\overset{-}{S}$-$\rho_{d}$− UCS correlation for low-density reef limestone was plotted (Figure 9).

It can be found from Figure 9 that when the density $\rho_{d}$ is fixed, the UCS increases with the reduction in $\overset{-}{S}$. When $\overset{-}{S}$ is fixed, UCS increases with increasing density $\rho_{d}$. The modified form of Equation (6) was used to fit the image data, giving:

(7) $$\mathrm{UCS}=(2.88-9.32\rho_{d})\mathrm{lg}\overset{-}{S},\;R^{2}=0.91$$

The degree of fit in Equation (6) is 0.91, showing a good degree of fit. In comparison with analysis results for the UCS correlation, the use of Equation (6) for the pore geometric parameter $\overset{-}{S}$ and density demonstrates good fitting performance, indicating that the influences of density and pore structures should be considered when analyzing the factors influencing the UCS of low-density reef limestones. According to testing results obtained, the UCS of low-density reef limestones is influenced by the combined effects of the pore geometric parameter $\overset{-}{S}$ and density. The UCS increases with the decrease in $\overset{-}{S}$, and grows with the enhancement in density.

## 4. Discussion

The mechanical properties of rock are generally closely related to factors including loading rate, rock type, and density. Previous research into the factors influencing the UCS of reef limestone shows that the influence of pore structure is negligible owing to the large density of the specimens, so relevant conclusions can be obtained by only considering the basic physical parameters of rock. Low-density reef limestone exhibits the significant feature of being a porous material, and the influence of pore structure on its mechanical properties is worthy of investigation.

In the present work, an image recognition technique was used to acquire geometric data of pore structures for low-density reef limestone. By defining pore geometric parameter $\overset{-}{S}$, the influence of pore structure on the UCS was quantified. It was concluded that the UCS was jointly influenced by the pore geometric parameter $\overset{-}{S}$ and the density. The results enrich the understanding of the factors influencing the UCS of low-density reef limestone, and provide an explanation for the special phenomenon found by Burton et al. [7]. 

Recently, Wei et al. [25] analyzed the coupled effects of particle size and porosity of coral aggregates and established a multi-factor model of the compressive strength of coral aggregates based on the principle of separation of variables, as shown in Equation (8):

(8) $$f_{d,p}=\lambda_{d,p}\left[exp\left(-d/\alpha_{d,p,1}\right)+C_{d,p,1}\right]\cdot \left[exp\left(-p/\alpha_{d,p,2}\right)+C_{d,p,2}\right]$$

where $f_{d,p}$ is the compressive strength, *d* is the particle size, *p* is the porosity, and $\lambda_{d,p}$, $\alpha_{d,p,1}$, $\alpha_{d,p,2}$, $C_{d,p,1}$, $\alpha_{d,p,2}$ are fitting coefficients.

Equation (6) is similar to Equation (8) in derivation process and form and also fits better than the single-factor analysis model. This suggests that it is feasible to utilize an image recognition technique to investigate the characteristics of pore geometry and investigate the mechanical properties of low-density reef limestone based on the theory of the mechanics of porous media.

Furthermore, the physico-mechanical properties, relative density, and pore structure of skeletal materials also influence the mechanical properties of porous materials [40]. The limitations of this research are that only a simplified analysis of pore geometry and experimental investigation of the factors influencing the UCS of low-density reef limestone were conducted without considering the differences in the physico-mechanical properties of skeletal materials. Hence, future research will be conducted based on the detailed characterization of the physico-mechanical properties and pore geometric characteristics of skeleton materials to explore the mechanical properties of low-density reef limestone and relevant constitutive models.

## 5. Conclusions

The UCS of low-density reef limestone was investigated using image recognition techniques. Conclusions can be drawn as follows:

(1) The pore structure of low-density reef limestone is significant: different types of pore structures demonstrate significant differences in terms of their shapes and sizes. By means of image recognition techniques, 2D geometric data pertaining to the pores within low-density reef limestone can be readily acquired. Through using unified photographic methods and comparing them with manual identification results, batch identification of the porous structure of specimens can be realized by optimizing the parameters involved in the image recognition process and image recognition results.
(2) Low-density reef limestones with different porous structures show small difference in porosity and density, while they exhibit large differences in pore sizes and UCS.
(3) By introducing the pore geometric parameter $\overset{-}{S}$, a multi-factor model of the UCS of low-density reef limestone was established, which fits better than the single-factor analysis model.

(4) The UCS of low-density reef limestones is influenced by the combined effects of the pore geometric parameter $\overset{-}{S}$ and density. The UCS increases with the decrease in $\overset{-}{S}$ and grows with the enhancement in density.

(5) The image recognition technology and data optimization method in this paper have significance for the rapid estimation of the strength of low-density reef limestone in engineering practice.

## Figures and Tables

**Table 1:** The application field of image recognition technique.

Application Field	References
Intelligent excavation	[29]
Assessing the roughness coefficients of rock joints	[30]
Measurement of local deformation in rock and soil	[31]
Identification of tunnel lining water leakage	[32]
Quantification of ceramsite granules in lightweight concrete panels	[33]
Detection of rockfill gradation	[34]

**Table 2:** $\overset{-}{S}$ values calculated based on different data sources.

Data Sources	Manual Recognition	The Raw Data of Image Recognition	The Optimized Data of Image Recognition
Total pore area (mm^2^)	17.465	21.166	18.356
Number of pores	93	241	71
$\overset{-}{S}$ (mm^2^)	0.1878	0.0878	0.2585

**Table 3:** Test results.

Sample Number	Porosity (%)	$\mathrm{Density}\;\rho_{d}$ (g/cm^3^)	$\overset{-}{S}$ (mm^2^)	UCS (MPa)
S-1	63.29	1.02	0.424	1.964
S-2	64.15	0.96	0.417	2.654
S-3	50.48	1.35	0.394	3.457
S-4	71.79	0.76	0.266	1.612
S-5	66.83	0.93	0.315	4.730
S-6	56.90	1.02	0.304	1.467
S-7	54.96	0.97	0.359	1.732
M-1	59.46	1.14	0.040	10.952
M-2	48.16	1.44	0.039	15.160
M-3	64.59	0.99	0.032	7.686
M-4	65.61	0.94	0.030	11.918
M-5	63.16	0.94	0.033	7.958

## Data Availability

The data presented in this study are available on request from the corresponding author.
